# Supplementary material for: SwiftReg cluster registration automatically reduces flow cytometry data variability including batch effects
Source: Commun Biol. 2020 May 7;3:218. doi: 10.1038/s42003-020-0938-9 (PMC7205614; doi:10.1038/s42003-020-0938-9)
Supplement: Supplementary file 1 — Supplementary Information [file 42003_2020_938_MOESM1_ESM.pdf]

Supplementary Information:

Title: swiftReg cluster registration automatically reduces flow cytometry data variability including batch effects.

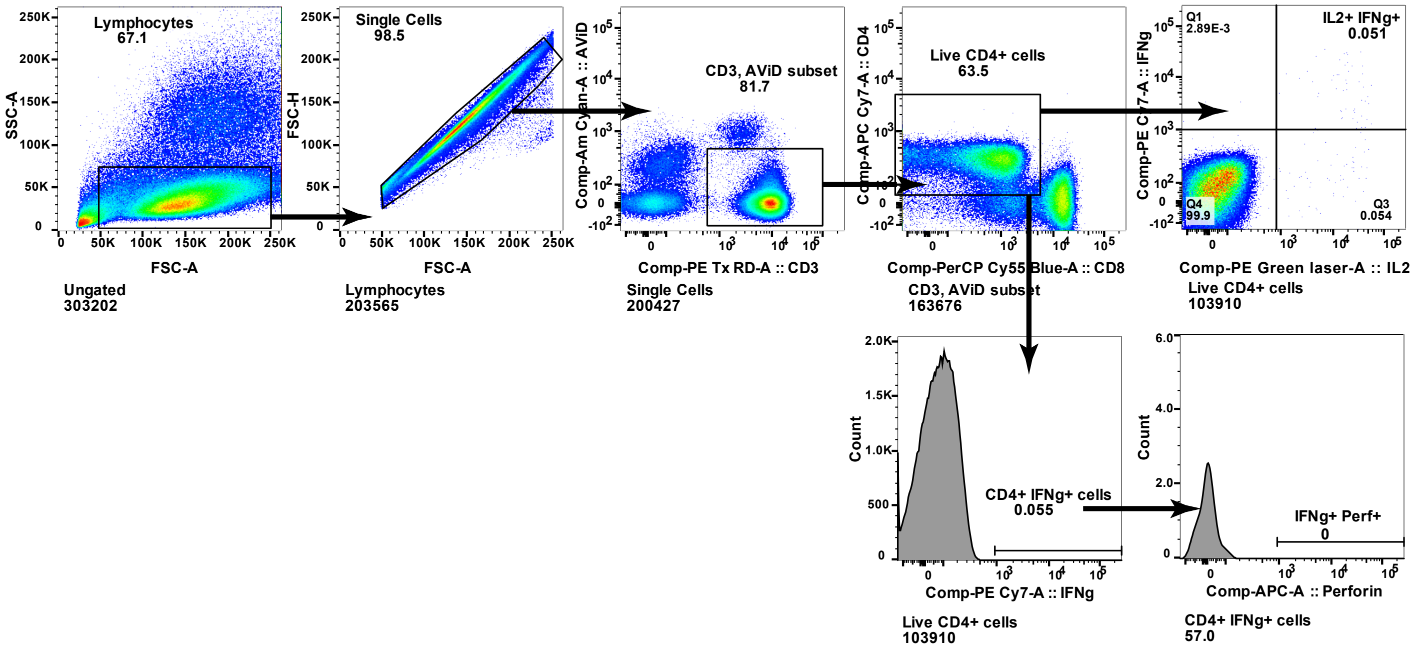

Supplementary Figure 1
Gating strategy for sub-populations of HVTN080 samples.

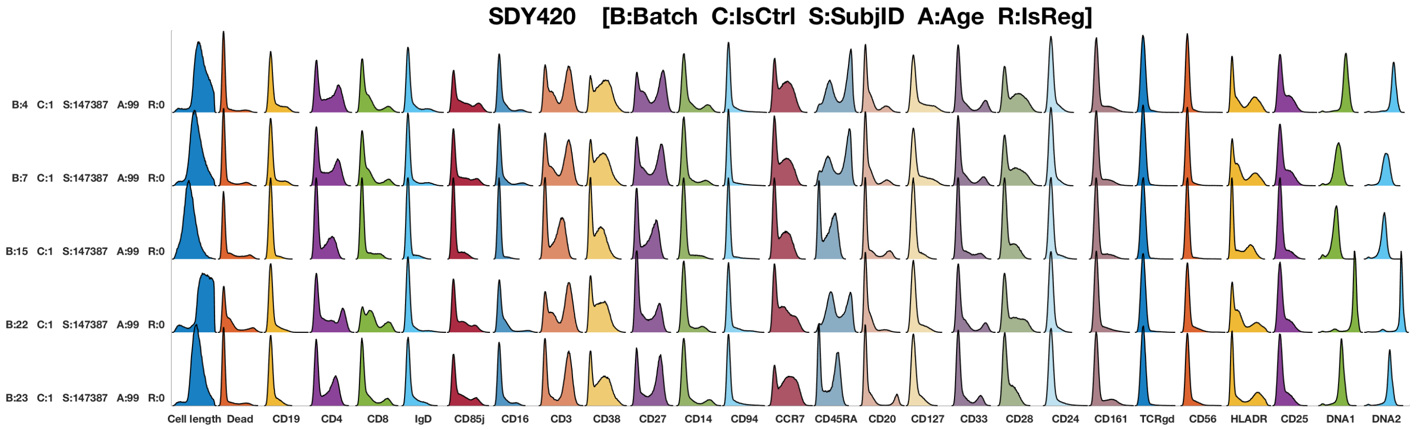

Supplementary Figure 2
Ungated histograms of all channels (columns) are shown for **non-registered** internal standard control samples (rows) of five batches from the SDY420 dataset.

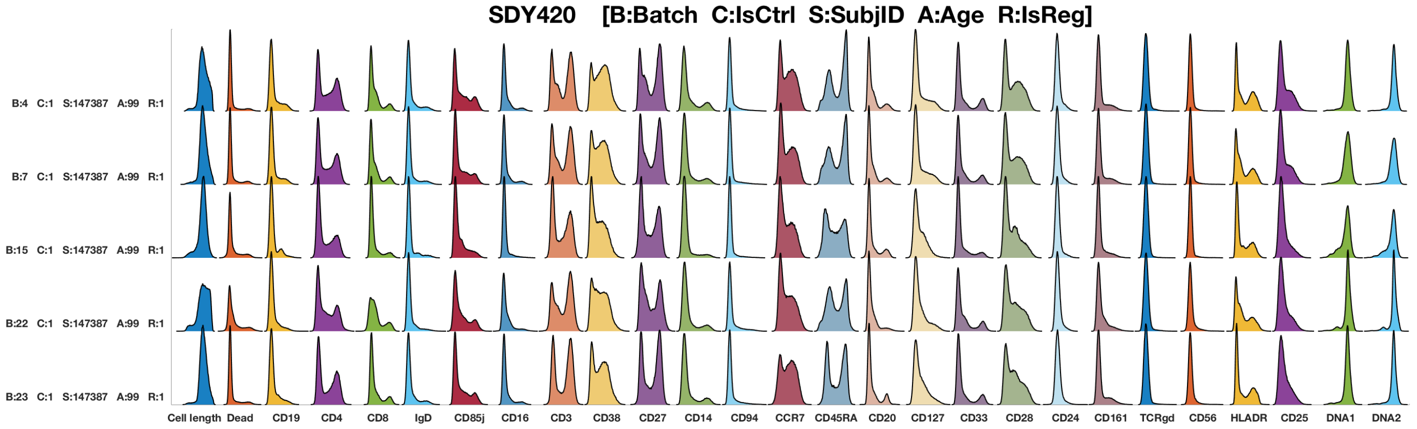

Supplementary Figure 3
The same samples as in Supplementary Figure 2 were registered to the internal standard from batch 4, and ungated histograms (as in Supplementary Figure 2) are shown for the **registered** internal standards.

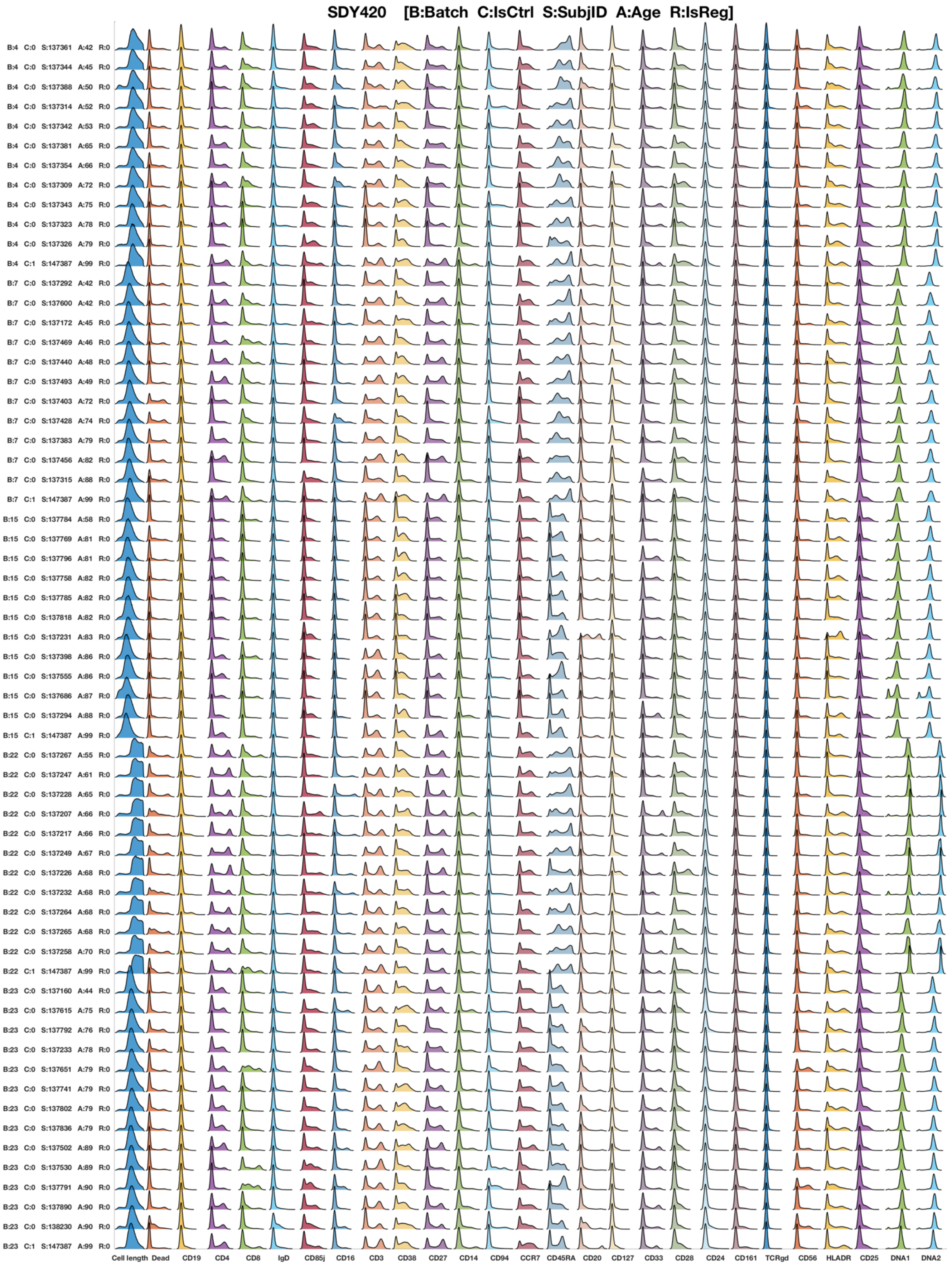

Supplementary Figure 4

Ungated histograms (as in Supplementary Figure 2) are shown for all **non-registered** samples from the same five batches.

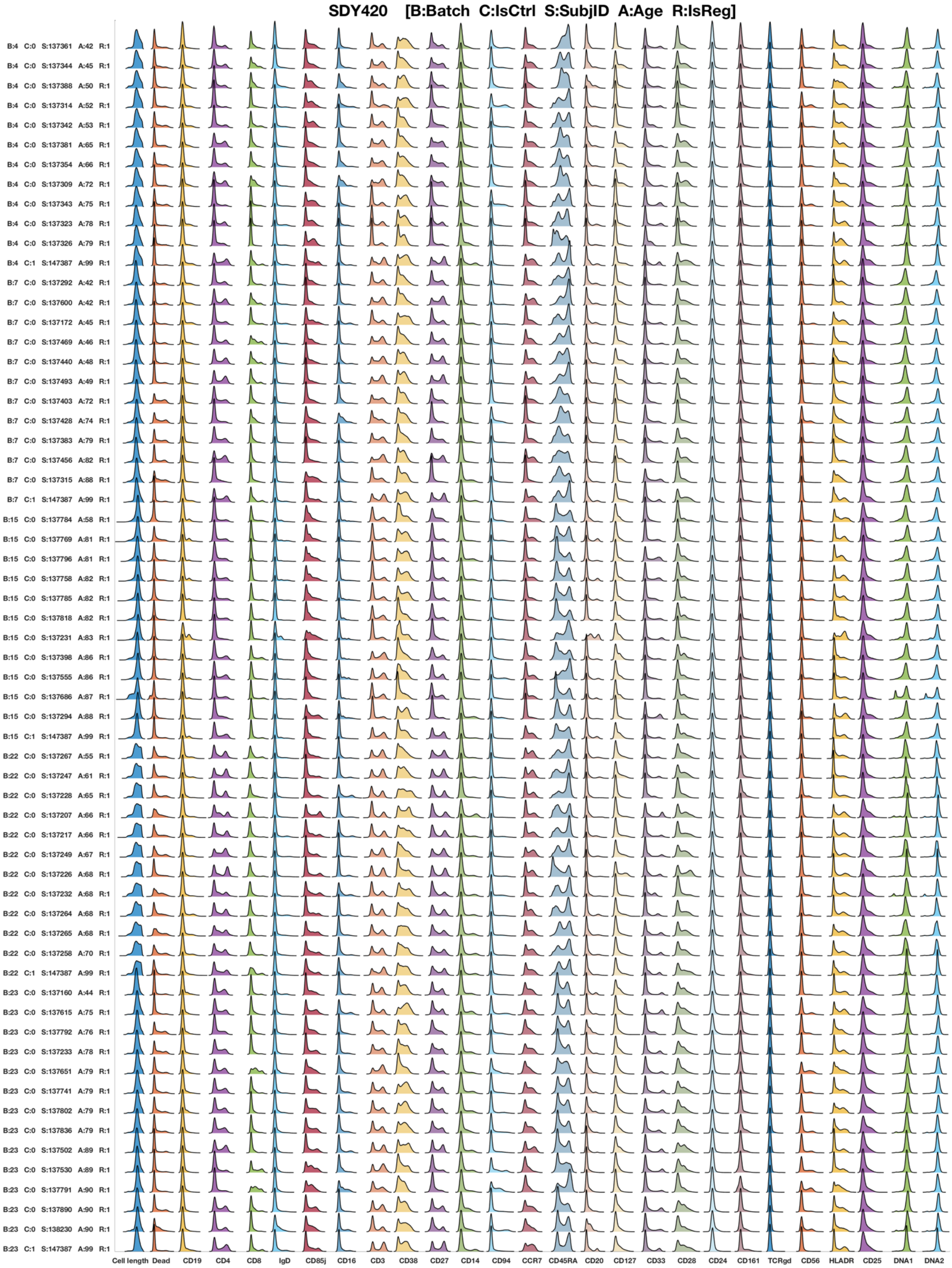

Supplementary Figure 5

Ungated histograms are shown for the same samples as in Supplementary Figure 4 after batch-registration to their respective internal standard registration templates.

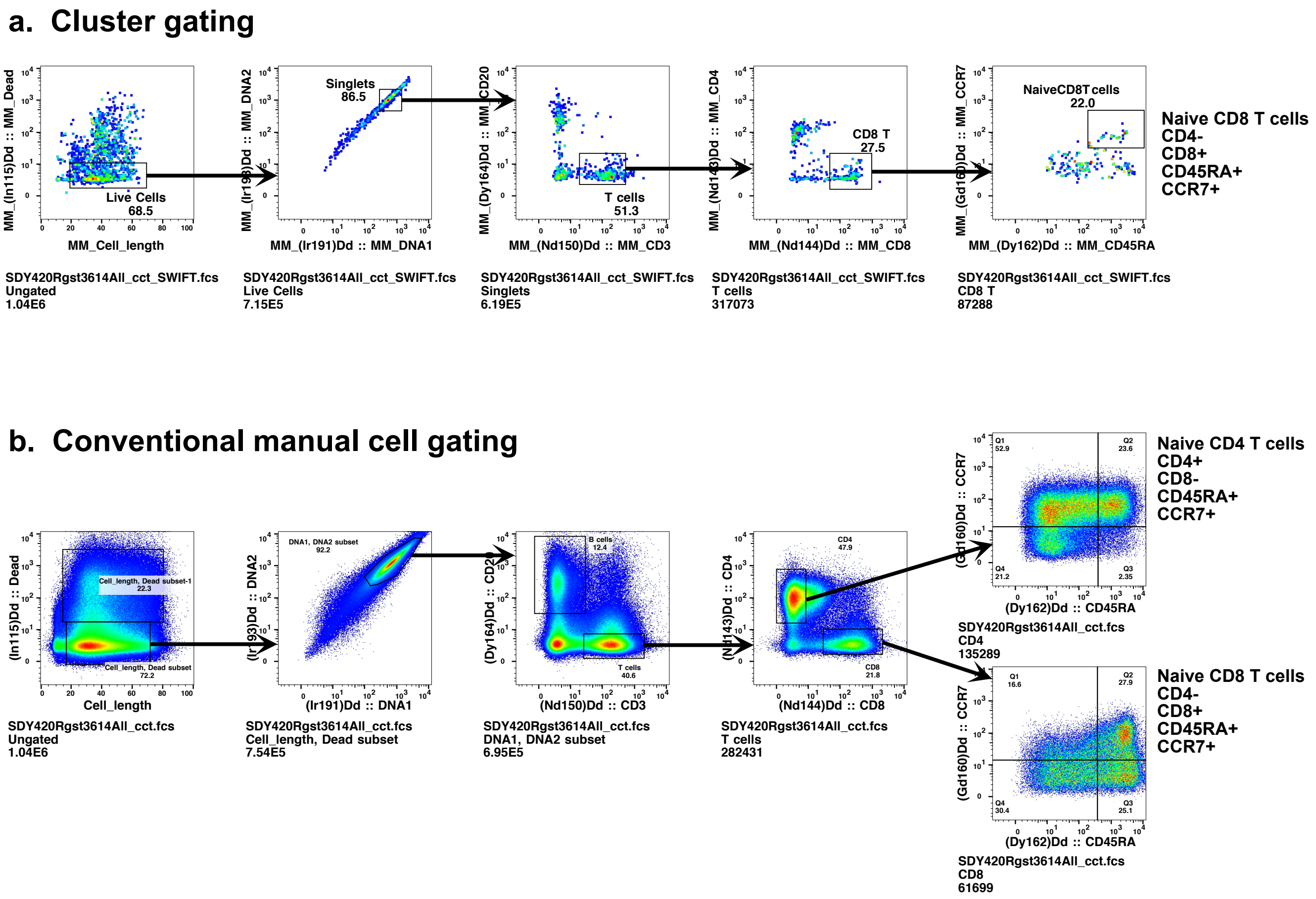

Supplementary Figure 6

(a). Cluster gating strategy for SDY420. All cells in a concatenate of all registered SDY420 samples (see Figure 8) were plotted at the median values of their cluster, allowing conventional FlowJo gating to operate on whole clusters. Sequential gating of the naïve CD8 T cell clusters is shown.

(b). Manual gating strategy for SDY420. All cells in a concatenate of all registered SDY420 samples (see Figure 9) were gated by conventional FlowJo gating, to isolate the naïve CD4 and naïve CD8 T cell populations.

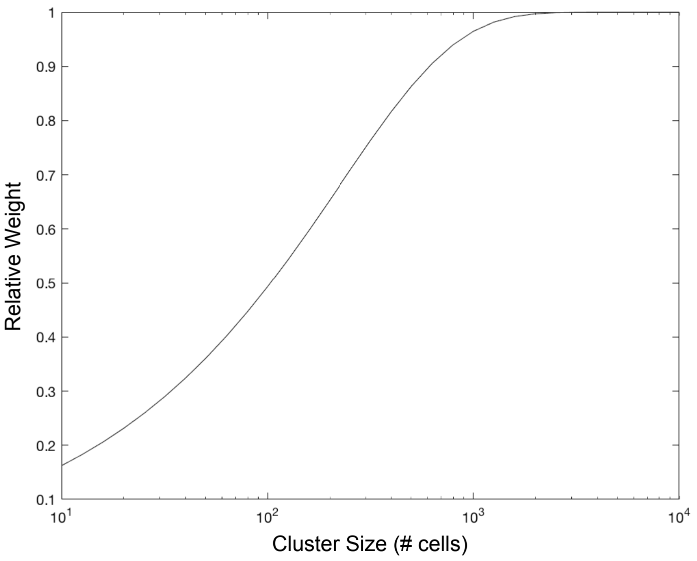

Supplementary Figure 7

Default cluster weights (y-axis) relative to cluster size (x-axis) are shown for determining the best-fit correction line for NDCR registration. The cluster size is the minimum size of a test sample cluster and its corresponding reference cluster. A probability is calculated from the Student's t cumulative distribution function with degrees of freedom given by the cluster size. The cluster weight is based on the probability that a sample cluster is correctly assigned to its cluster in the reference.

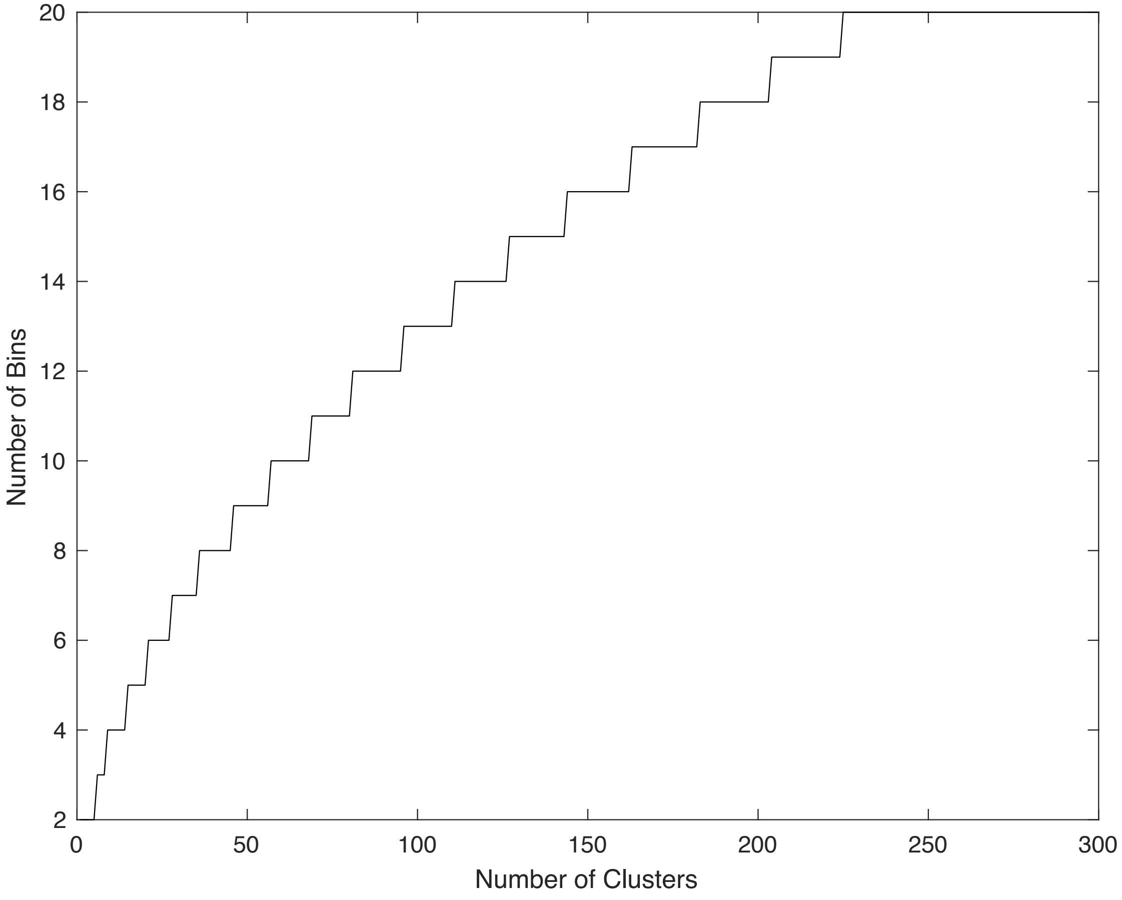

Supplementary Figure 8
The number of bins used (y-axis) relative to the number of clusters (x-axis) are shown for determining the best-fit correction line for NDCR registration. The minimum and maximum number of bins is constrained to 2 and 20, respectively.
