# Supplementary material for: SwiftReg cluster registration automatically reduces flow cytometry data variability including batch effects
Source: Commun Biol. 2020 May 7;3:218. doi: 10.1038/s42003-020-0938-9 (PMC7205614; doi:10.1038/s42003-020-0938-9)
Supplement: Supplementary file 2 — Reporting Summary [file 42003_2020_938_MOESM2_ESM.pdf]

nature research

Corresponding author(s): Tim Mosmann

Last updated by author(s): Mar 26, 2020

Reporting Summary

Nature Research wishes to improve the reproducibility of the work that we publish. This form provides structure for consistency and transparency in reporting. For further information on Nature Research policies, see Authors & Referees and the Editorial Policy Checklist.

Statistics

For all statistical analyses, confirm that the following items are present in the figure legend, table legend, main text, or Methods section.

n/a	Confirmed	
☐	☒	The exact sample size (*n*) for each experimental group/condition, given as a discrete number and unit of measurement
☐	☒	A statement on whether measurements were taken from distinct samples or whether the same sample was measured repeatedly
☐	☒	The statistical test(s) used AND whether they are one- or two-sided<br>*Only common tests should be described solely by name; describe more complex techniques in the Methods section.*
☒	☐	A description of all covariates tested
☐	☒	A description of any assumptions or corrections, such as tests of normality and adjustment for multiple comparisons
☐	☒	A full description of the statistical parameters including central tendency (e.g. means) or other basic estimates (e.g. regression coefficient) AND variation (e.g. standard deviation) or associated estimates of uncertainty (e.g. confidence intervals)
☐	☒	For null hypothesis testing, the test statistic (e.g. *F*, *t*, *r*) with confidence intervals, effect sizes, degrees of freedom and *P* value noted<br>*Give P values as exact values whenever suitable.*
☒	☐	For Bayesian analysis, information on the choice of priors and Markov chain Monte Carlo settings
☒	☐	For hierarchical and complex designs, identification of the appropriate level for tests and full reporting of outcomes
☐	☒	Estimates of effect sizes (e.g. Cohen's *d*, Pearson's *r*), indicating how they were calculated

Our web collection on statistics for biologists contains articles on many of the points above.

Software and code

Policy information about availability of computer code

Data collection	No primary data were collected in this study - data from public repositories was used.
Data analysis	The SWIFT clustering and registration package was used. This software is publicly available for download at:<br>http://www.ece.rochester.edu/projects/siplab/Software/SWIFT.html<br>Commercial FlowJo V10 software was used for manual analysis.

For manuscripts utilizing custom algorithms or software that are central to the research but not yet described in published literature, software must be made available to editors/reviewers. We strongly encourage code deposition in a community repository (e.g. GitHub). See the Nature Research guidelines for submitting code & software for further information.

Data

Policy information about availability of data

All manuscripts must include a data availability statement. This statement should provide the following information, where applicable:

- Accession codes, unique identifiers, or web links for publicly available datasets
- A list of figures that have associated raw data
- A description of any restrictions on data availability

The datasets analyzed during the current study are available in the following repositories/Accession Codes:
JMW090 (8 samples) https://flowrepository.org/id/FR-FCM-ZZ8W
JMW090 (40 samples) https://flowrepository.org/id/FR-FCM-Z284
JMW092 https://flowrepository.org/id/FR-FCM-Z283
HVTN080 https://flowrepository.org/id/FR-FCM-ZZ7U
SDY420 https://www.immport.org/shared/study/SDY420

Field-specific reporting

Please select the one below that is the best fit for your research. If you are not sure, read the appropriate sections before making your selection.

☒ Life sciences ☐ Behavioural & social sciences ☐ Ecological, evolutionary & environmental sciences

For a reference copy of the document with all sections, see nature.com/documents/nr-reporting-summary-flat.pdf

Life sciences study design

All studies must disclose on these points even when the disclosure is negative.

Sample size	Publicly available datasets were used. Sample size determinations were performed by the authors of the individual studies.
Data exclusions	All available samples were used in each study, except:<br>Figures 1 and 4, six subjects, influenza-stimulated samples only, were selected arbitrarily to illustrate dataset variability.<br>Figure 3ab, three samples, and Figure 3c, four samples were chosen arbitrarily to test the registration parameters.<br>Figure 5, panel b shows data from all samples. Panel a shows detailed data from batches 880 and 1053 (1053 was the batch with the highest content of aberrant cells).<br>Figure 7, all the internal batch standards are shown, plus one selected batch to demonstrate modifications in actual samples.<br>Figure 8,9, out of 260 subjects, the oldest 20 and the youngest 20 were used for statistical comparisons. Detailed data from all subjects are shown.
Replication	In all figures analyzing actual, not model data (except figure 5), multiple datasets were analyzed, with concordant results. In the analysis shown in Figure 5, one dataset was used because this was the only dataset used in the paper describing a previous registration method. Several independent registration analyses were performed using other batches as reference, and the results were concordant.
Randomization	Publicly available datasets were used. In figure 5, subject allocation was decided in the original study. In Figure 8-9, the oldest 20 and youngest 20 were chosen for comparisons. Other comparisons (e.g. oldest 30 vs youngest 30) gave similar results.
Blinding	Publicly available datasets were used. Samples were not blinded during method development and analysis.

Reporting for specific materials, systems and methods

We require information from authors about some types of materials, experimental systems and methods used in many studies. Here, indicate whether each material, system or method listed is relevant to your study. If you are not sure if a list item applies to your research, read the appropriate section before selecting a response.

Materials & experimental systems

n/a	Involved in the study
☒	☐ Antibodies
☒	☐ Eukaryotic cell lines
☒	☐ Palaeontology
☒	☐ Animals and other organisms
☒	☐ Human research participants
☐	☒ Clinical data

Methods

n/a	Involved in the study
☒	☐ ChIP-seq
☐	☒ Flow cytometry
☒	☐ MRI-based neuroimaging

Clinical data

Policy information about clinical studies

All manuscripts should comply with the ICMJE guidelines for publication of clinical research and a completed CONSORT checklist must be included with all submissions.

Clinical trial registration	Publicly available datasets were used, i.e. no primary clinical studies were performed.
Study protocol	Publicly available datasets were used, i.e. no primary clinical studies were performed.
Data collection	Publicly available datasets were used, i.e. no primary clinical studies were performed.
Outcomes	Publicly available datasets were used, i.e. no primary clinical studies were performed.

Flow Cytometry

Plots

Confirm that:

☒ The axis labels state the marker and fluorochrome used (e.g. CD4-FITC).

☒ The axis scales are clearly visible. Include numbers along axes only for bottom left plot of group (a 'group' is an analysis of identical markers).

☒ All plots are contour plots with outliers or pseudocolor plots.

☒ A numerical value for number of cells or percentage (with statistics) is provided.

Methodology

Sample preparation	Publicly available datasets were used, i.e. no primary clinical studies were performed.
Instrument	Publicly available datasets were used, i.e. no primary clinical studies were performed.
Software	The SWIFT clustering and registration package was used. This software is publicly available for download at:<br>http://www.ece.rochester.edu/projects/siplab/Software/SWIFT.html<br>Commercial FlowJo V10 software was used for manual analysis.
Cell population abundance	No sorting was performed.
Gating strategy	Gating strategies are shown in the supplemental data.

☒ Tick this box to confirm that a figure exemplifying the gating strategy is provided in the Supplementary Information.
